# GWAS, QTL mapping and gene expression analyses in *Brassica napus* reveal genetic control of branching morphogenesis

**DOI:** 10.1038/s41598-017-15976-4

**Published:** 2017-11-21

**Authors:** Yajun He, Daoming Wu, Dayong Wei, Ying Fu, Yixin Cui, Hongli Dong, Chuandong Tan, Wei Qian

**Affiliations:** College of Agronomy and Biotechnology, Southwest University, Chongqing, 400716 China

## Abstract

Branch number is an important trait in plant architecture that can influence crop yield and quality in *Brassica napus*. Here, we detected the QTLs responsible for branch number in a DH population and its reconstructed F_2_ population over two years. Further, a GWAS research on branch number was performed using a panel of 327 accessions with 33186 genomic SNPs from the 60 K Brassica Illumina® Infinium SNP array. Through combining linkage analysis and association mapping, a new QTL was fine mapped onto C03. Subsequently, we tested the correlations between the SNP polymorphisms and mRNA expression levels of genes in the target interval to identify potential loci or genes that control branch number through expression. The results show that 4 SNP loci are associated with the corresponding gene expression levels, and one locus (BnaC03g63480D) exhibited a significant correlation between the phenotype variation and gene expression levels. Our results provide insights into the genetic basis for branching morphogenesis and may be valuable for optimizing architecture in rapeseed breeding.

## Introduction

The branch system is an important part of plant architecture^1–5^. The number of branches (BN) on the main stem is one of the most important properties in rapeseed architecture. A proper BN may aid in improving rapeseed yield and quality.

The shoot branching process generally involves two developmental stages, the formation of axillary meristems in the leaf axils and the growth of axillary buds^6–10^. This process is controlled by multiple factors and can be influenced by environmental conditions or developmental signals^1,7,9–22^. Three classical phytohormones, auxin, cytokinin, and strigolactone, and the genes associated with their homeostasis and signaling are likely largely responsible for regulating branching^1,21–23^. Moreover, other signals, such as sugars or molecular actors on plant phase transition, also strongly influence shoot branching^11^. To date, many genes involved in axillary meristem initiation and outgrowth have been reported in tomato, rice, *Arabidopsis*, maize, pea, petunia and barley^24–33^. These genes are mostly associated with homeostasis and signaling of phytohormones and growth regulators. In addition, certain transcription factors that play a role in regulating the transcription of other genes, such as *REV* ^34^, *LAS* ^35^ and *RAX 1*, 2, and 3^36^, also control branching. Moreover, the genes encoding cytochrome P450, MAPKK7, arabinogalactan proteins, and other DNA-binding proteins have been identified that are involved in branch outgrowth^37^.

Linkage mapping is well-established and has been employed for mapping QTLs to determine quantitative traits in rapeseed^38–45^. However, fine mapping QTLs using a linkage analysis requires a population with thousands of individuals, and the limited polymorphic loci between the two parents will influence the mapping accuracy^46^. Recently, association mapping has been widely employed to detect quantitative trait loci^47–51^. It directly identifies associations between DNA markers and phenotypes in natural populations based on linkage disequilibrium (LD)^52^. Compared with linkage mapping, association mapping does not require constructing special mapping populations, and it uses high recombination in natural populations. Therefore, association mapping can complement linkage mapping and facilitate fine-scale QTL mapping^52,53^. However, in an association analysis, the population structure can produce a stronger LD between non-linked loci due to genetic drift, population stratification and natural selection^54,55^. Thus, combining association mapping and a linkage analysis not only avoids the false positives from associated loci due to high LD but also facilitates fine mapping of a target region with a large QTL interval^56^.

In rapeseed, certain QTLs that are related to branch number have been identified through linkage analyses in recent years^57–61^. Most QTLs were located on chromosomes A02, A06, A07, C03, C06 and C09 and were only detected in a single-year environment. Recently, Luo *et al*.^62^ reported six SNPs on A03 associated with rapeseed branch number using genome-wide association study (GWAS). However, despite the studies on rapeseed branching, the basis for genetically controlling branching morphogenesis has not been fully elucidated for rapeseed, and few genes have been predicted in the QTL intervals. Better understanding the genetic determinants for rapeseed branching is needed.

In the present study, the QTLs responsible for branch number were detected in a doubled haploid (DH) population and its reconstructed F_2_ (RC-F_2_) population. Further, a GWAS research on branch number was performed using a panel of 327 accessions with 33186 genomic SNPs from the 60 K *Brassica* Illumina^®^ Infinium SNP array. Combined with the gene expression analysis, a candidate gene that controls the rapeseed branch number was identified.

## Results

### Phenotypic variation in DH, RC-F_2_ and association populations

**Figure 1:**
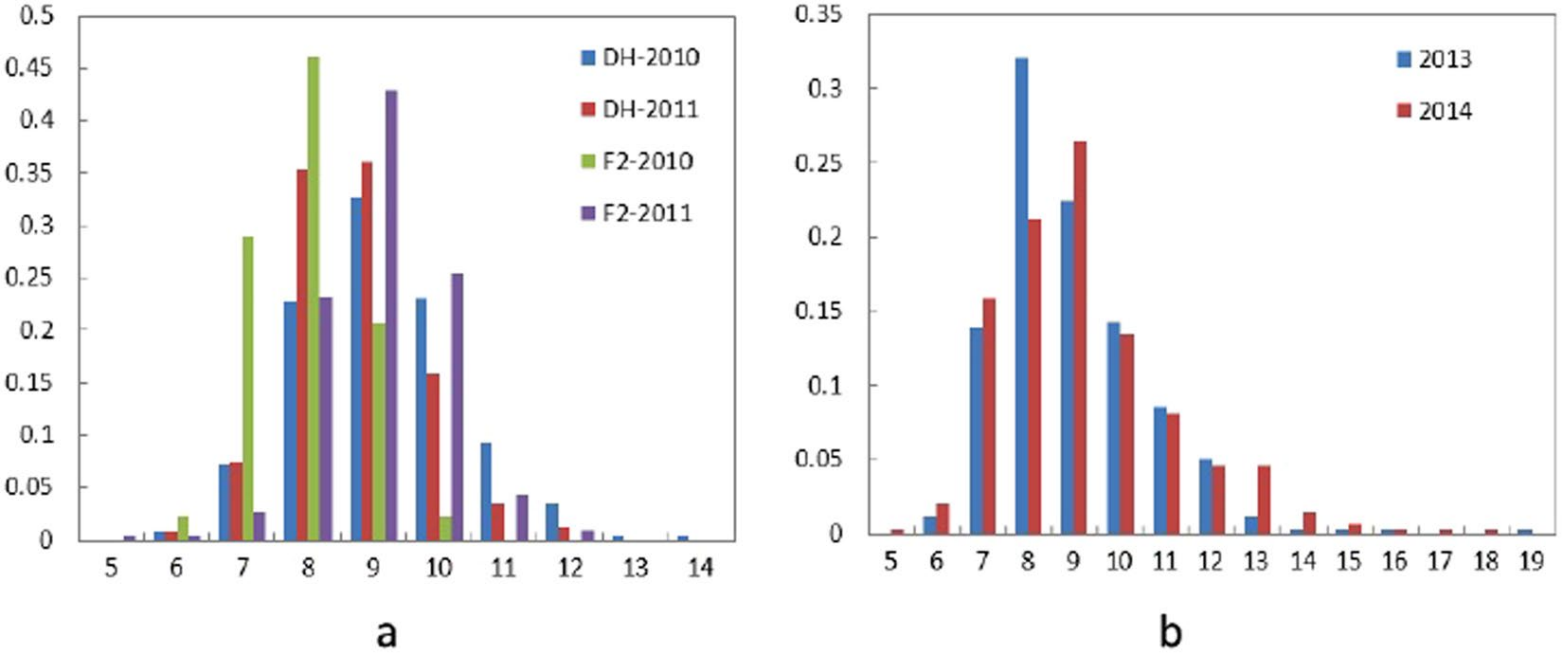
Phenotype frequency distribution of BN in the mapping populations. (**a**) DH and RC-F_2_ population; (**b**) association mapping panel.

The average branch number of the parental line ‘SWU07’ is 7.92, whereas that of the other parental line ‘Express’ is 8.50. Extensive phenotypic variations for DH, RC-F_2_ and association mapping populations used in this study were observed (Fig. 1, Table 1). The frequency distribution of BN for the three populations appears to be an approximately normal distribution in all the separate experiments, which indicated that the branch number phenotype is governed by multiple genes (Fig. 1, Table S1). Transgressive segregation was observed in the DH and RC-F_2_ populations, which suggests that the loci control BN was harbored by different alleles of the two parents.

**Table 1:** Phenotypic variations for BN in DH, RC-F_2_ and association mapping populations.

Population	MIN	MAX	MEAN ± SD^a^	CV%^b^
DH-2010	6	13.25	8.78 ± 1.26	14.33%
DH-2011	5.75	11.25	8.30 ± 0.99	11.91%
RC-F_2_ -2010	5.38	9.88	7.49 ± 0.77	10.23%
RC-F_2_ -2011	5	12	8.62 ± 0.93	10.80%
Association panel -2013	5.6	18.8	8.66 ± 2.21	25.48%
Association panel -2014	5	17.1	9.57 ± 2.70	28.18%

^a^SD is an abbreviation of standard deviation. ^b^CV is an abbreviation of coefficient of variation, which was estimated as the ratio of the standard deviation to the mean of all accessions.

The results of ANOVA revealed significant differences among genotypes, environments and genotype-by-environment interactions for branch number in the three populations (P < 0.01) (Table 2). High broad-sense heritability was detected for BN with average of 49.19%, 67.02% and 79.17% in DH, RC-F_2_ and association mapping population, respectively (Table 2). The significant and positive correlation for BN was detected between the years in the three populations (r = 0.52, 0.28 and 0.59 in the DH, RC-F_2_ and association mapping populations, respectively; P < 0.01).

**Table 2:** Analysis of variance and heritability for BN in DH, RC-F_2_ and association mapping populations.

Population	Source	Df	Mean Square	Heritability (%)
DH	Genotype	260	2.4233	49.19
	Year	1	0.3691	
	Genotype × Year	243	0.7992	
	Genotype	232	0.9991	
RC-F_2_	Year	1	114.7237	67.02
	Genotype × Year	178	0.5077	
	Genotype	326	4.7420	
Association panel	Year	1	30.6939	79.17
	Genotype × Year	213	0.98754	

### QTL analysis

**Figure 2:**
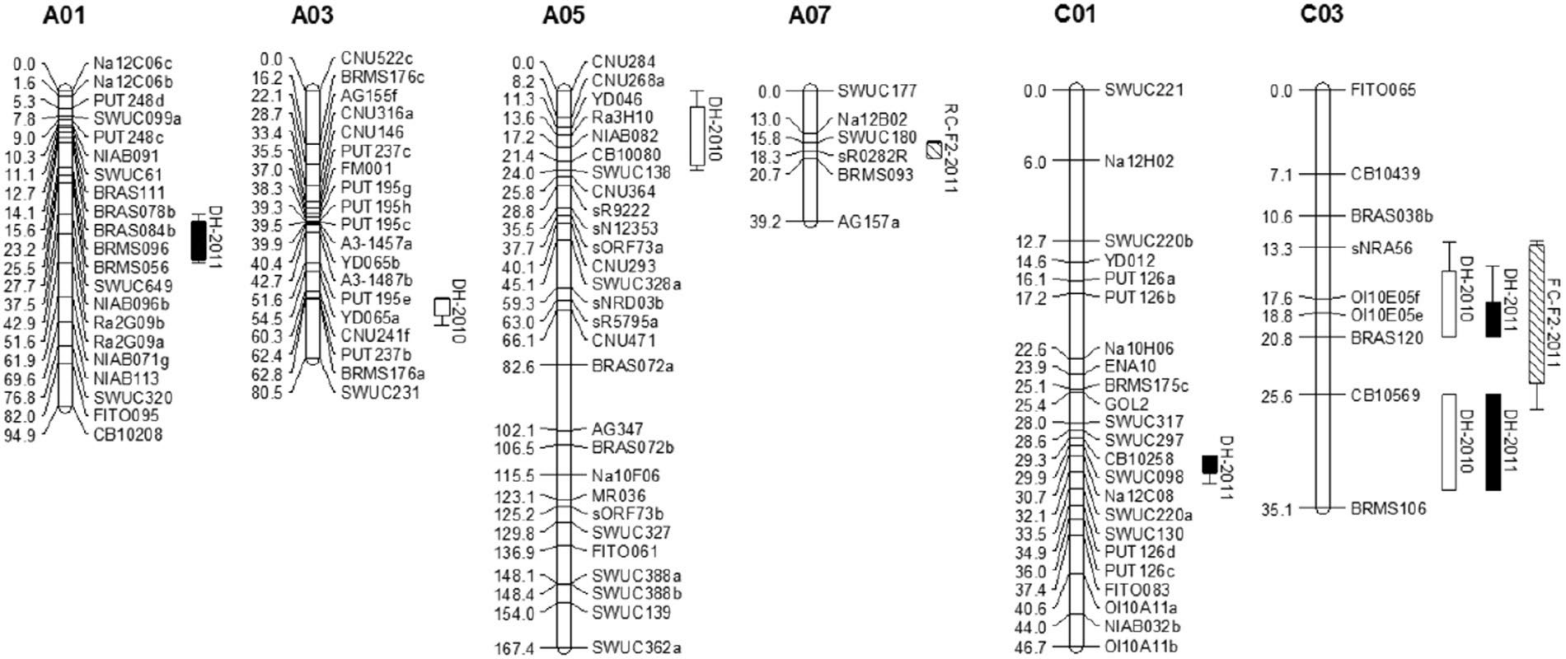
QTL for BN in the DH and RC-F_2_ populations in 2010 and 2011.

Ten QTLs associated with BN are located on chromosomes A01, A03, A05, A07, C01 and C03 in the DH and RC-F_2_ populations over the two years, which explained 3.73–9.77% of the phenotypic variation (Fig. 2, Table 3). Two QTLs on C03 were detected in different years or in different populations. Finally, seven QTLs were generated after integrating the QTLs with overlapped confidence intervals in different experiments.

**Table 3:** QTLs for BN in DH and RC-F_2_ populations.

Pop.	Envi.	Chr.	Flanking markers	LOD	Add	R^2^ (%)	Confidence interval
DH	2010	A03	PUT237b~SWUC231	2.74	0.28	3.97	62.5~70.8
DH	2010	A05	CNU284~CNU364	3.10	0.29	4.86	0.00~24.0
DH	2010	C03	BRAS038b~BRAS120	2.55	−0.26	3.73	12.9~20.8
DH	2010	C03	CB10569~BRMS106	3.49	−0.37	6.41	25.6~33.6
DH	2011	A01	NIAB096b~Ra2G09a	2.72	0.30	5.15	37.5~51.6
DH	2011	C01	Na12C08~SWUC130	3.10	−0.37	8.05	30.7~33.1
DH	2011	C03	sNRA56~BRAS120	2.67	−0.28	4.14	14.9~20.8
DH	2011	C03	CB10569~BRMS106	2.70	−0.35	5.56	25.6~33.6
RC-F_2_	2011	A07	BRMS093~BRMS093	3.11	−0.26	4.97	15.2~20.6
RC-F_2_	2011	C03	Ol10E05f~CB10569	5.90	−0.31	9.77	12.7~26.8

### Population structure and relative kinship in association population

**Figure 3:**
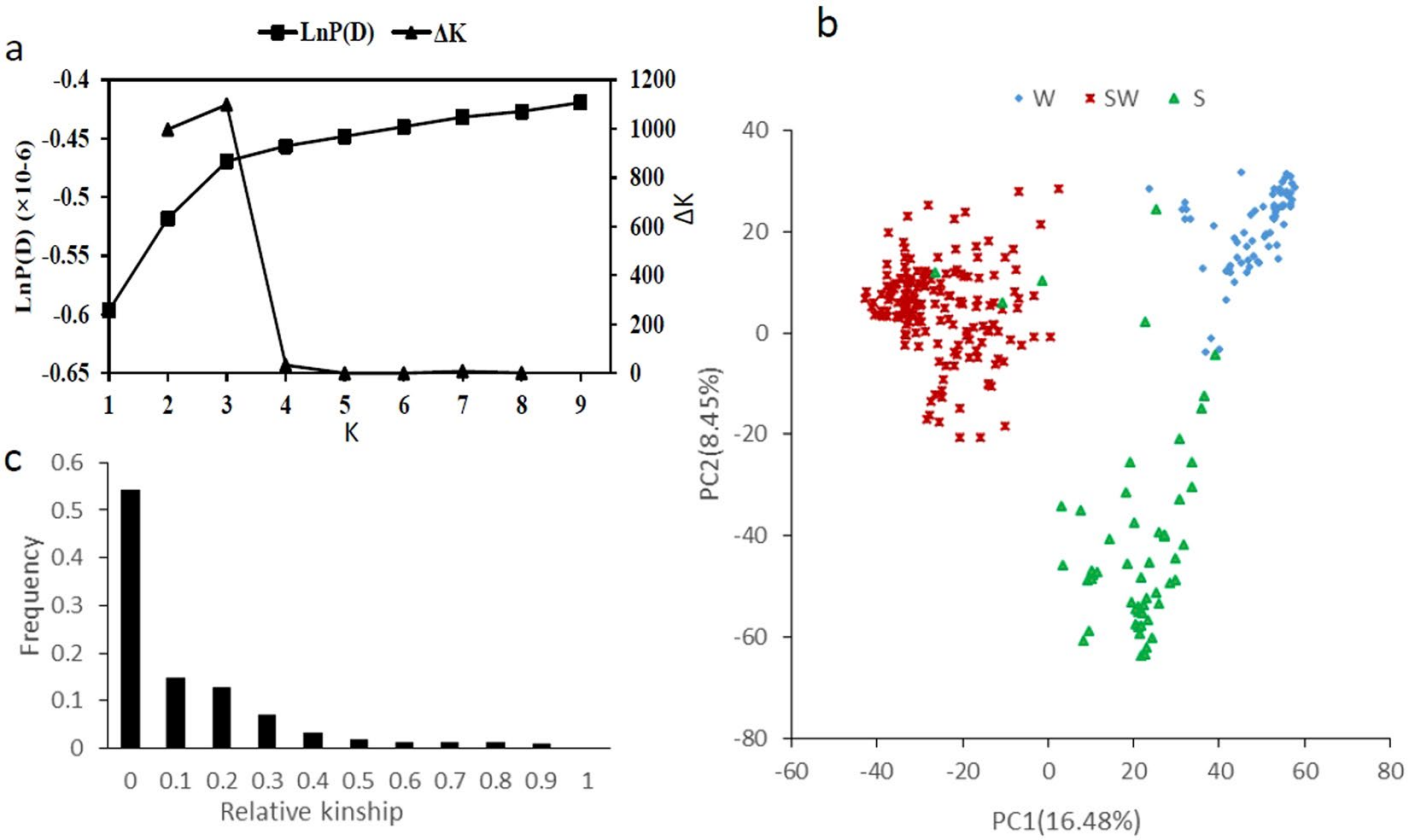
Analysis of population structure and relative kinship in 327 *B. napus*. (**a**) Estimated LnP(D) for K from 1 to 10 and ΔK between successive K; (**b**) Plots of the first two principal components (PC1 and PC2); (**c**) Distribution of relative kinship values.

The population structure was estimated using 5700 SNP. We select 300 SNP in each chromosome of *B. napus*. The result showed that the most significant change of likelihood occurred when K increased from 3 to 4, and the highest Δk value was observed at k = 3 (Fig. 3a). The two parameters suggested that the 327 genotypes were assigned into three groups. The PCA based on the 33,186 genome-wide SNPs showed that the first two principal components explained 16.48 and 8.45% of the genetic variance, respectively (Fig. 3b). The 327 accessions were classified into three major groups, which are mainly composed of winter ecotype, spring ecotype, and semi-winter ecotype, respectively (Fig. 3b). It is largely in accordance with the growth habit of the accessions. We used 33,186 polymorphisms to estimate the relative kinship of the materials. The analysis showed that total of 54% of kinship coefficients between lines were equal to 0, and 69% kinship coefficients were less than 0.1(Fig. 3c). This pattern of genetic relatedness revealed that most lines have no or weak kinship in this association panel.

### Genome-wide association analysis

**Figure 4:**
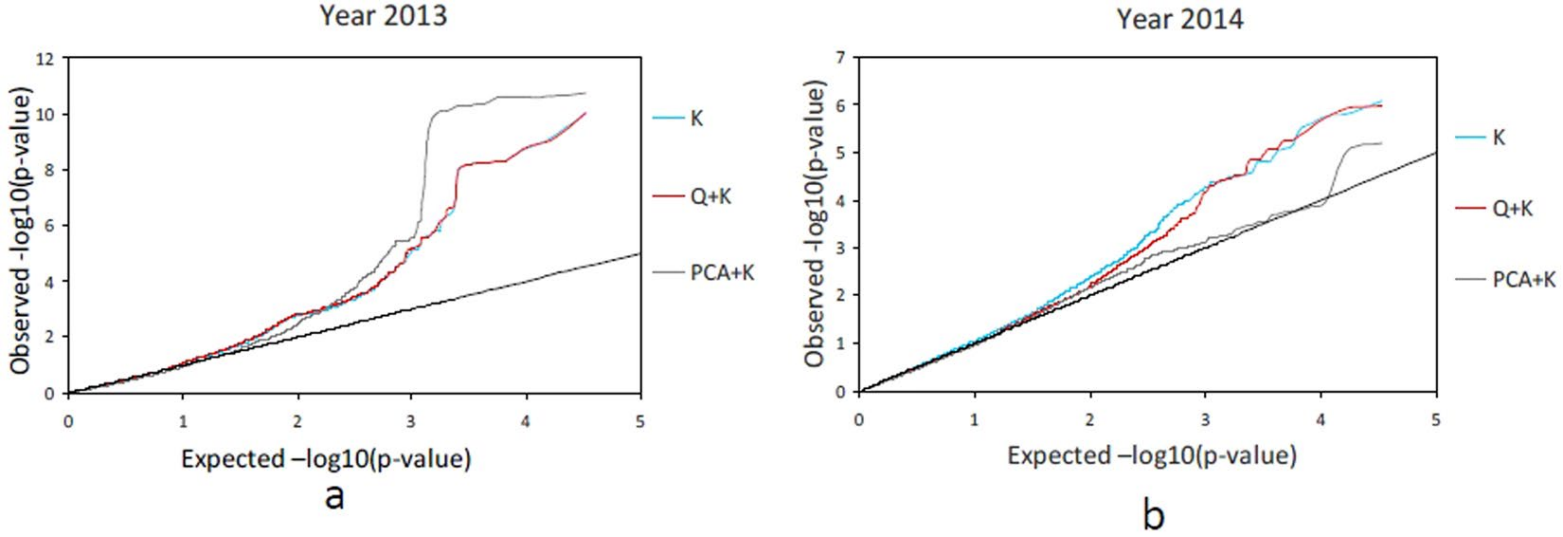
Quantile–quantile plots of K model, Q + K model and PCA + K model in two years. (**a**) In the year of 2013; (**b**) In the year of 2014. The black line is the expected line under the null distribution.

Since the dataset used in the association study consists of three subgroups, winter, semi-winter and spring ecotype, the relative kinship is weak. However, it still has a certain impact for the GWAS analysis. To avoid the false negative associations, three mixed models, K model, Q + K model and PCA + K model were chosen to determine the statistical associations between phenotypes and genotypes to evaluate the effects of population structure (Q, PC) and relative kinship (K) on BN traits. According to the Q-Q plots of the three models, for the 2013 data, the observed *P* values of the Q + K model were closer to the expected *P* values than the PCA + K model and K modle (Fig. 4a), whereas for the 2014 data, the observed P values of the PCA + K model were closer to the expected P values than the other two modles (Fig. 4b). This indicated that the Q + K model and PCA + K model could effectively control false positive associations and avoid false negative associations for 2013 and 2014 data, respectively. Thus, we choose Q + K and PCA + K models to perform the association analysis for the 2013 and 2014 data, respectively.

We used 33,186 polymorphisms with a minor allele frequency (MAF) ≥ 0.05 for a GWAS. Notable positive associations were observed in the Manhattan plots (Fig. S1). The association analysis identified 50 SNP loci significantly associated with branch number at P < 3.01 × 10^−5^ over the two years (Table S2; Fig. S1). 44 and 15 SNPs were detected in the year of 2013 and 2014, respectively, and 9 were detected in the two-year experiments. According to the decay of LD, the physically closed SNPs were integrated to the single QTL interval. Totally, four GWAS-based QTL regions were generated in the two-year experiments (Table 4), which explain 8.65–20.03% of the phenotypic variance.

**Table 4:** GWAS-based QTLs for BN in two-year experiments.

Chr.	QTL interval (Mb)	Envi.	p value	−LOG_10_(p)	R^2^ (%)
A02	15.29~15.59	2013	9.48E-11	10.02	20.03
		2014	1.06E-06	5.97	10.26
C03	27.39~28.89	2013	4.88E-09	8.31	16.40
		2014	5.77E-06	5.24	9.14
C03	52.19~53.69	2013	2.64E-06	5.58	10.68
		2014	1.39E-05	4.86	8.65
C09	45.11~46.62	2013	6.61E-09	8.18	17.03
		2014	8.36E-06	5.08	9.02

### Candidate Gene Prediction using GWAS, QTL mapping and gene expression analyses

**Figure 5:**
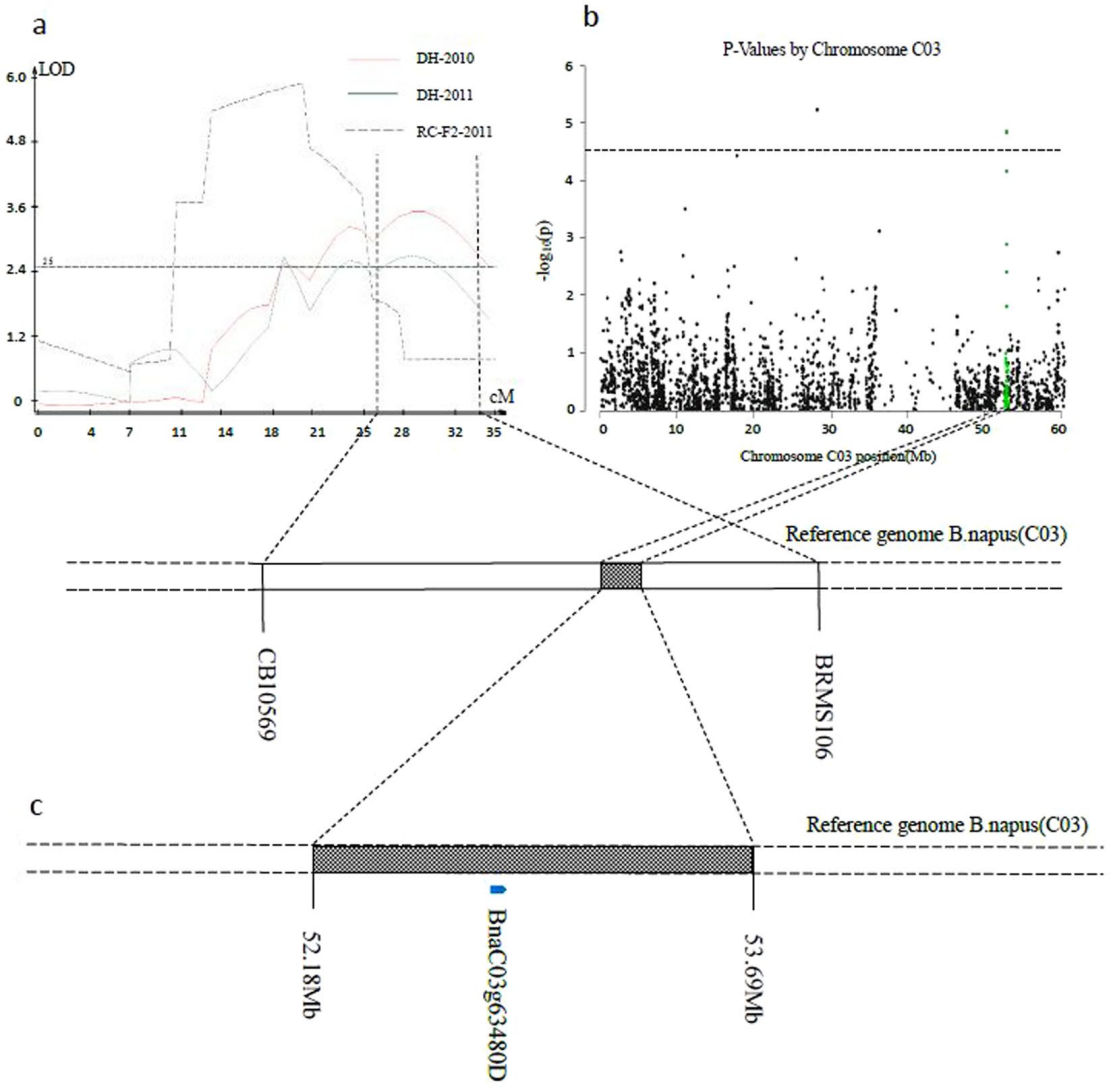
Identification of the target region controlling branch number by Linkage mapping and association mapping. (**a**) QTls associated with branch number detected on Chromosome C03; (**b**) Manhattanplots of association analysis for branch number. Each dot represents a SNP. The significance threshold −log10(p) = 4.52; (**c**) The target interval on Chromosome C03. The blue bar represents the gene which was identified to exhibit a significant correlation between phenotype variation of branch number and gene expression levels (at P < 0.05).

We compared the linkage mapping and association mapping results. Notably, one QTL interval on C03 was detected by the two mapping approaches. Four consecutive SNPs not only exhibited a significant correlation between DNA sequence polymorphisms and BN phenotypic variation in the GWAS analysis over the two years but were also located in the C03 QTL confidence interval by linkage mapping (Fig. 5a,b). This QTL were considered stably inherited. Therefore, we focused on the genes in this interval. The corresponding genomic sequences of the QTL region were extracted. An annotation analysis showed that chromosome C03 from 52. 18–53.69 Mb contains 138 genes with 125 SNP loci (Fig. 5c).

In the association mapping panel of this study, 31 inbred lines overlapped with the plant materials in a previous report by Bancroft *et al*.^63^. The RNA-seq data on the 31 inbred lines are available in an online resource^63^. Thus, we tested the correlations between the 125 SNP polymorphisms and mRNA expression levels of the 138 genes in the target interval to discern potential loci or genes that control branch number at the expression level. The results show that 4 of the 125 detected SNP loci, SNP46529, SNP50286, SNP50314, and SNP50315 are associated with the corresponding gene expression levels (Table S3). These 4 loci are positioned upstream region of 3 corresponding genes. SNP46529 and SNP50286 are positioned upstream of BnaC03g63340D and BnaC03g63530D, respectively. SNP50314 and SNP50315 are positioned upstream of BnaC03g63480D. The 4 SNP variation correlated (p < 0.01) with the corresponding gene expression levels at r = 0.5067 (SNP46529), r = −0.6142 (SNP50286), r = 0.5209 (SNP50314), and r = 0.5800 (SNP50315), respectively. Subsequently, we tested the correlations between phenotype variation and mRNA expression level of the three genes. Interestingly, among the three genes that showed a significant correlations between the SNP variation and gene expression levels, one gene (BnaC03g63480D) exhibited a significant correlation between the phenotype variation and gene expression level (at P < 0.05), while the other two have no correlation with the phenotype variation (P > 0.05). The gene expression levels of BnaC03g63480D correlated (p < 0.05) with the branch number at r = 0.1143 and r = 0.2679 in 2013 and 2014, respectively. These data strongly suggest that the gene BnaC03g63480D affects phenotypic variation via transcriptional regulation and it is a potential candidate gene for rapeseed branch number.

## Discussion

Branch number is an important trait in plant architecture that can influence crop yield and quality^1–5^. Identifying the genetic loci for branch number would aid in understanding the heredity mechanism underlying branching morphogenesis and be valuable in optimizing the architecture for rapeseed breeding. Previous research shows that environmental factors can influence branching, such as planting density^12,13^, photoperiod^9,10,14,15^, and nutrient availability^19,20^. Thus, multi-environment testing is necessary to determine whether the effects of QTLs are due to different genes or environments. Here, we detected numerous distinct loci that influence branch number in a DH population and its reconstructed F_2_ population over two years. We also detected the association SNP loci via GWAS in the two-year environments. The QTL regions which detected by both linkage mapping and association mapping were considered stably inherited. Notably, most QTLs or SNP loci were only detected in a single-year environment, which suggests that environmental variation plays an important role in determining rapeseed branching morphogenesis. The concerted effects of genotype, environment and their interactions determine branching morphogenesis.

Along with the development of high-throughput SNP genotyping technology, genome-wide association studies (GWAS) have been broadly applied to unravel the genetic basis for complex traits in many crops, such as maize^50^, rice^64^ and sorghum^65^. Association mapping can be used to finely map linkage analysis results when a QTL region is large, and linkage mapping can exclude false positives from associated loci due to high linkage disequilibrium. The combination of association mapping and linkage analysis can increase mapping efficiency and accuracy^56^. In the present study, we preliminarily mapped the QTLs responsible for branch number through linkage mapping in rapeseed. Subsequently, a GWAS analysis was performed to quickly verify the QTL region and finely map the QTL within a 1.51 Mb interval (52. 18–53.69 Mb) containing 138 genes on C03. The GWAS analysis reduced the target region size and will aid in further identifying the candidate gene. Compared with previous studies for linkage mapping and GWAS analyses on rapeseed branch number^57–62^, the QTL we detected here is a new one.

Genetic mechanisms that regulate phenotypic variation can act not only on a gene structure level but also on a gene expression level. The transcript sequence data for *Brassica napus* would aid in analyzing the correlations between DNA sequence polymorphisms and mRNA expression levels. We tested the correlations between SNPs and mRNA expression levels for the genes in the target interval. We also examined the correlations between branch number phenotype and mRNA expression level of these genes. Finally, BnaC03g63480D was identified as a candidate gene according statistical correlations. The structure and function of the candidate gene were analyzed. BnaC03g63480D encodes a putative ubiquitin E3 ligase containing a RING domain. A previous study showed that the RING domain interacts with other proteins^66^. In Arabidopsis, BOI (AT4G19700), the BnaC03g63480D homologs, interact with DELLA proteins to inhibit GA responses by interacting with each other, binding to the same promoters of GA-responsive genes, and repressing these genes^67^. Gibberellins play an important role in internode elongation^68^, but their role in shoot branching process has not been clearly elucidated. Previous studies show that GA-deficient mutants had higher shoot branching than the wild type in Arabidopsis^69^, rice^70^, and pea^71^, and over expressing GA catabolism genes to reduce GA levels can increase a branching phenotype^70,72^. No direct evidence shows that the RING domain proteins are related to branch formation, but the RING domain can repress GA signals has been reported^67^. Our data confirm that the expression level of the RING domain gene BnaC03g63480D significantly correlates with branch number. We speculate that BnaC063480D controls branching morphogenesis by regulating the GA-responsive gene in rapeseed. Further studies should investigate and confirm the possible function of BnaC03g63480D in rapeseed.

## Methods

### Mapping population and phenotypic evaluation

The DH and RC-F_2_ populations used in the QTL analysis were previously described in Fu *et al*.^73^. The DH population consisting of 261 lines was developed by microspore culture, using a single F_1_ plant derived from a cross between the European winter oilseed rape cultivar ‘Express’ (female) and the Chinese semi-winter inbred line ‘SWU07’ (male). The RC-F_2_ population consisting of 233 lines was generated by two rounds of random crosses between DH lines. Each DH line was used once each round. The two populations and the parental lines were planted in the experimental field of Southwest University, Chongqing, China, in 2010 and 2011. A randomised complete block design with two replications was employed.

The association mapping panel used in the GWAS analysis was composed of 327 diverse inbred lines, including 71 winter ecotypes from Europe, 196 Chinese semi-winter accessions and 60 spring ecotype lines randomly selected from the ERANET-ASSYST *B. napus* diversity set. These lines were grown in the experimental field of Southwest University, Chongqing, China, in 2013 and 2014, and two replications were planted for each line in each year.

The branch number for ten representative *B. napus* plants in each line of the mapping populations were measured at maturity. Analysis of variance (ANOVA) was performed using the GLM procedure of SAS^74^.

### QTL analysis

Development of molecular markers and construction of genetic linkage groups was described in a previous study^73^, where 293 markers were mapped to 19 linkage groups with a 1,188 cM map distance. Detection of QTLs and estimation of genetic parameters were performed with composite interval mapping (CIM) procedure of the software WinQTL Cartographer version 2.5^75^. The QTLs were declared significant if the corresponding LR score was greater than 11.5 (equal to a 2.5 LOD score). The percent of phenotypic variance (PV) explained by a QTL (R^2^) was estimated at the highest probability peaks. A permutation test was performed 1,000 times at a significance level of p = 0.005 to minimize the experimental type-I error rate.

### Genome-wide association analysis

The association mapping panel of 327 inbred lines was genotyped using the *Brassica* 60 K Illumina^®^ Infinium SNP array by Emei Tongde Co. (Beijing) in accordance with the manufacturer’s protocol (http://www.illumina.com/technology/beadarray-technology/infinium-hd-assay.html). We excluded SNPs with either an AA or a BB frequency equal to zero, call frequency <0.9, or minor frequency <0.05.

Population structure was analyzed using the software package STRUCTURE v2.3.4^76^. Five independent runs were performed with a K-value (the putative number of genetic groups) from 1 to 10, with 10,000 MCMC (Markov chain Monte Carlo) replications and 10,000 burn-ins. The optimal k-value was determined by the log probability of data [LnP(D)] and an ad hoc statistic Δk based on the rate of change of LnP(D) between successive k^77^. The software package SPAGeDi v1.4 was employed to calculate the relative kinship matrix comparing all pairs of the 327 accessions^78^. Negative values between two individuals were set to 0^79^.

To avoid the false negative associations caused by relative kinship, the three mixed models controlling relative kinship, K model, controlling for K; Q + K model, controlling for both Q and K; and PCA + K model, controlling for both PC and K, were chosen to determine the statistical associations between phenotypes and genotypes to evaluate the effects of population structure (Q, PC) and relative kinship (K) on BN traits. These three models were performed with optimum compression and population parameters previously determined (P3D) by variance component estimation in TASSEL 5.0^79,80^. Statistically significant loci were identified by comparing P values with the Bonferroni threshold (1/33186 = 3.01E-5)^62^.

### Gene expression analysis

Thirty-one inbred lines of the association mapping panel in this study overlapped with the plant materials in a previous report by Bancroft *et al*.^63^. The RNA-seq data are available through an online resource. To determine whether the expression levels of genes in the target interval identified through QTL mapping and a GWAS analysis was associated with the BN observed in this study, the correlations between the gene expression levels and the SNP variations within or upstream of the corresponding genes were analyzed. The correlations between the BN phenotype variation and gene expression levels were also tested. Statistical analysis was completed using *t* test and correlation analysis.

### Candidate gene predication

The QTL intervals were aligned to the *B. napus* reference genomes (http://www.genoscope.cns.fr/blat-server/cgi-bin/colza/webBlat) by BLAST the sequences of the SSR markers linked with BN QTLs. Based on the physical flanking marker positions of the QTLs, the corresponding genomic sequences of the QTL region were extracted. According to our previously study (Wei *et al*., data not published), the LD decay is 150 Kb in A subgenome and 750 kb in C subgenome. According to the decay of LD, the confident region of the GWAS-based QTL were determined. The QTL regions which identified by both linkage mapping and association mapping were selected and considered stably inherited. The corresponding genomic sequences of the region were extracted. The genes in the interval were used for a gene expression analysis. Finally, the correlations between the BN phenotype and the genes expression levels were examined to determine the candidate genes that control branch number.

## Electronic supplementary material

Supplementary information

Supplementary Dataset 1
