# Supplementary material for: GWAS, QTL mapping and gene expression analyses in Brassica napus reveal genetic control of branching morphogenesis
Source: Sci Rep. 2017 Nov 21;7:15971. doi: 10.1038/s41598-017-15976-4 (PMC5698412; doi:10.1038/s41598-017-15976-4)
Supplement: Supplementary file 1 — Supplementary information [file 41598_2017_15976_MOESM1_ESM.pdf]

**GWAS, QTL mapping and gene expression analyses in *Brassica napus* reveal genetic control of branching morphogenesis**

Yajun He, Daoming Wu, Dayong Wei, Ying Fu, Yixin Cui, Hongli Dong, Chuandong Tan, Wei Qian*

College of Agronomy and Biotechnology, Southwest University, Chongqing 400716, China

Yajun He and Daoming Wu contributed equally to this work

*Corresponding author
Phone: +86 (0) 23 68250701
Fax: +86 (0) 23 68250701
qianwei666@hotmail.com

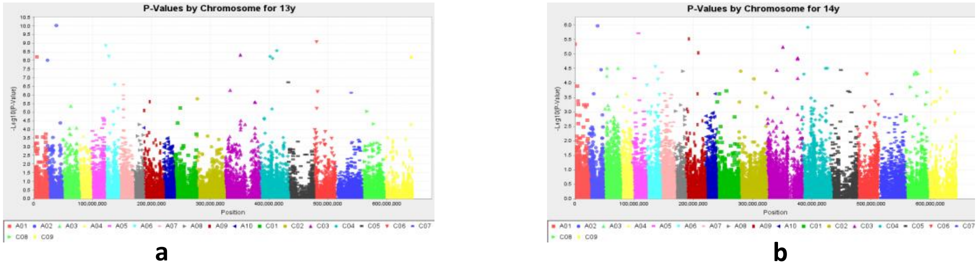


a b

Figure S1. Manhattanplots of association analysis for branch number. Each dot represents a SNP. The horizontal dashed line represents the Bonferroni-corrected significance threshold -log10 ( p) = 4.52. (a) In the year of 2013; (b) In the year of 2014.

Table S1. Normality test for distributions of BN in the mapping populations

| Shapiro-wilk | Branch number(MS) | | | | | |
|---|---|---|---|---|---|---|
| | GWAS population | | DH Population | | RC-F2 population | |
| year | 2013 | 2014 | 2010 | 2011 | 2010 | 2011 |
| W | 0.8917 | 0.9228 | 0.9859 | 0.9717 | 0.9948 | 0.9846 |
| P-Value(Pr<W) | <0.0001 | <0.0001 | 0.0115 | <0.0001 | 0.7871 | 0.0128 |

Table S2. Genome-wide significant association signals detected in 2013 and 2014, respectively

| Year | SNP | Chr. | Site | p | $-LOG_{10}(p)$ | $R^2$ |
|---|---|---|---|---|---|---|
| 2013 | SNP2154 | A01 | 5154600 | 6.08E-09 | 8.215782242 | 0.16979 |
| 2013 | SNP4177 | A02 | 362847 | 9.65E-09 | 8.015652742 | 0.17559 |
| 2013 | SNP31355* | A02 | 15444754 | 9.48E-11 | 10.02313669 | 0.2003 |
| 2013 | SNP5809 | A03 | 15121067 | 4.29E-06 | 5.367107619 | 0.10607 |
| 2013 | SNP11815 | A05 | 21057548 | 2.17E-05 | 4.662980247 | 0.09057 |
| 2013 | SNP11942 | A05 | 22822603 | 2.57E-05 | 4.590337339 | 0.11233 |
| 2013 | SNP13220 | A06 | 17886136 | 2.62E-07 | 6.581765018 | 0.1273 |
| 2013 | SNP13555 | A06 | 15584464 | 9.36E-06 | 5.028700952 | 0.12461 |
| 2013 | SNP14029 | A06 | 23362511 | 5.96E-06 | 5.22473188 | 0.10168 |
| 2013 | SNP14198 | A06 | 2730311 | 1.44E-09 | 8.840162209 | 0.17775 |
| 2013 | SNP14911 | A06 | 7976724 | 5.82E-09 | 8.234927799 | 0.16538 |
| 2013 | SNP17039 | A07 | 8173848 | 1.06E-06 | 5.975596437 | 0.11826 |
| 2013 | SNP17085 | A07 | 8484782 | 1.78E-06 | 5.748970461 | 0.11115 |
| 2013 | SNP17089 | A07 | 8486061 | 6.96E-06 | 5.157690377 | 0.10568 |
| 2013 | SNP17092 | A07 | 8487016 | 6.51E-06 | 5.186345635 | 0.10644 |
| 2013 | SNP17093 | A07 | 8488014 | 2.46E-07 | 6.608694312 | 0.12885 |
| 2013 | SNP17101 | A07 | 8519521 | 6.96E-06 | 5.157690377 | 0.10568 |
| 2013 | SNP17090 | A07 | 8486172 | 2.03E-05 | 4.691713111 | 0.09568 |
| 2013 | SNP1660 | A09 | 866809 | 7.96E-06 | 5.099026921 | 0.11009 |
| 2013 | SNP19612 | A09 | 10038891 | 2.40E-06 | 5.619228157 | 0.11183 |
| 2013 | SNP38204 | C01 | 10961783 | 5.52E-06 | 5.258257658 | 0.12484 |
| 2013 | SNP48312 | C02 | 1028041 | 1.66E-06 | 5.780782343 | 0.13731 |
| 2013 | SNP28989* | C03 | 28143619 | 4.88E-09 | 8.31165138 | 0.16402 |
| 2013 | SNP28991* | C03 | 28144397 | 4.88E-09 | 8.31165138 | 0.16402 |
| 2013 | SNP42624 | C03 | 28790966 | 2.99E-05 | 4.524125511 | 0.12075 |
| 2013 | SNP49229 | C03 | 10830449 | 5.32E-07 | 6.273802742 | 0.1258 |
| 2013 | SNP50325* | C03 | 52936681 | 2.64E-06 | 5.578412524 | 0.10684 |
| 2013 | SNP50326* | C03 | 52937164 | 2.64E-06 | 5.578412524 | 0.10684 |
| 2013 | SNP50329* | C03 | 52938677 | 2.60E-06 | 5.585143593 | 0.107 |
| 2013 | SNP50335* | C03 | 52941999 | 2.58E-06 | 5.589138446 | 0.10703 |
| 2013 | SNP30456 | C04 | 22394199 | 7.75E-09 | 8.110754339 | 0.17262 |
| 2013 | SNP32798 | C04 | 18239032 | 5.90E-09 | 8.228890432 | 0.16839 |
| 2013 | SNP33406 | C04 | 21377746 | 6.34E-06 | 5.197760067 | 0.10091 |
| 2013 | SNP47478 | C04 | 29470363 | 2.73E-09 | 8.563869171 | 0.17292 |
| 2013 | SNP49183 | C04 | 8168059 | 2.25E-05 | 4.6473352 | 0.09184 |
| 2013 | SNP49184 | C04 | 8179335 | 2.50E-05 | 4.602720621 | 0.09254 |
| 2013 | SNP32110 | C05 | 309242 | 1.86E-07 | 6.729927037 | 0.13177 |
| 2013 | SNP28053 | C06 | 7120761 | 6.58E-07 | 6.181562951 | 0.13276 |
| 2013 | SNP45473 | C06 | 5637714 | 5.98E-06 | 5.223211675 | 0.10355 |
| 2013 | SNP45481 | C06 | 5345708 | 8.26E-10 | 9.083072534 | 0.18757 |
| 2013 | SNP25662 | C07 | 27102106 | 7.29E-07 | 6.137093786 | 0.11838 |
| 2013 | SNP35012 | C08 | 9405456 | 8.86E-06 | 5.052546672 | 0.09575 |
| 2013 | SNP36475* | C09 | 45874293 | 6.61E-09 | 8.180002266 | 0.17031 |
| 2013 | SNP36479* | C09 | 45861679 | 6.61E-09 | 8.180002266 | 0.17031 |

| | | | | | | |
|---|---|---|---|---|---|---|
| 2014 | SNP2078 | A01 | 93843 | 4.48E-06 | 5.348692905 | 0.09854 |
| 2014 | SNP31355* | A02 | 15444754 | 1.06E-06 | 5.973711938 | 0.1026 |
| 2014 | SNP44470 | A05 | 10787455 | 1.91E-06 | 5.718466686 | 0.09795 |
| 2014 | SNP13085 | A06 | 16312688 | 2.76E-05 | 4.558902135 | 0.10525 |
| 2014 | SNP20287 | A09 | 21453018 | 9.05E-06 | 5.043125934 | 0.07251 |
| 2014 | SNP37577 | A09 | 5544826 | 3.02E-06 | 5.519317694 | 0.09436 |
| 2014 | SNP28989* | C03 | 28143619 | 5.77E-06 | 5.238568352 | 0.09135 |
| 2014 | SNP28991* | C03 | 28144397 | 5.77E-06 | 5.238568352 | 0.09135 |
| 2014 | SNP50325* | C03 | 52936681 | 1.39E-05 | 4.857986167 | 0.08652 |
| 2014 | SNP50326* | C03 | 52937164 | 1.39E-05 | 4.857986167 | 0.08652 |
| 2014 | SNP50329* | C03 | 52938677 | 1.40E-05 | 4.852509179 | 0.08639 |
| 2014 | SNP50335* | C03 | 52941999 | 1.47E-05 | 4.831384678 | 0.08559 |
| 2014 | SNP39307 | C04 | 9649769 | 1.18E-06 | 5.927529518 | 0.08751 |
| 2014 | SNP36475* | C09 | 45874293 | 8.36E-06 | 5.077726194 | 0.09019 |
| 2014 | SNP36479* | C09 | 45861679 | 8.36E-06 | 5.077726194 | 0.09019 |

* significant association signals both detected in the two-year environments

Table S3. Correlation analysis between SNP polymorphisms and gene expression levels

| SNP | BnaC03g63340D | | BnaC03g63480D | | BnaC03g63530D | |
|---|---|---|---|---|---|---|
| | Correlation | P value | Correlation | P value | Correlation | P value |
| SNP46529 | 0.5067 | 0.0452 | — | — | — | — |
| SNP50314 | — | — | 0.5209 | 0.0386 | — | — |
| SNP50315 | — | — | 0.5800 | 0.0185 | — | — |
| SNP50286 | — | — | — | — | -0.6142 | 0.0114 |

Note: SNP46529 is positioned upstream of BnaC03g63340D, SNP50314 and SNP50315 are positioned upstream of BnaC03g63480D, and SNP50286 is positioned upstream of BnaC03g63530D.
